# External-internal ureteral catheterization technique in treatment of ureteral injuries

**DOI:** 10.3906/sag-1902-3

**Published:** 2019-08-08

**Authors:** Hasanali DURMAZ

**Affiliations:** 1 Department of Radiology, Dışkapı Yıldırım Beyazıt Training and Research Hospital, University of Health Sciences, Ankara Turkey

**Keywords:** Ureter, injuries, catheterization

## Abstract

**Background/aim:**

It was aimed to describe the external-internal ureteral catheterization technique and evaluate its safety, efficacy, and reliability in iatrogenic and traumatic ureteral injuries.

**Materials and methods:**

A retrospective review was performed on patients with iatrogenic and traumatic ureteral injury, treated using the external-internal ureteral catheterization technique between May 2012 and January 2018 in our hospital. A total of 14 patients were investigated with clinical, postoperative, and follow-up findings, as well as technical outcomes.

**Results:**

The urology, gynecology, and general surgery departments referred patients for treatment at a rate of 57% (n = 8), 36% (n = 5), and 7% (n = 1), respectively. The causes were urological procedures for lithiasis (43%, n = 6), gynecological surgery (36%, n = 5), rectosigmoid surgery (7%, n = 1), penetrating injury (7%, n = 1), and partial nephrectomy (7%, n = 1). The most commonly affected segment was the distal third of the ureter, at a rate of 79% (n = 11). The mean duration of catheterization in all of the patients was 39 days. The overall technical success was 100% and no major complications occurred.

**Conclusion:**

The external-internal ureteral catheterization technique in patients with ureteral injury is easy to apply and effective not only in reducing costs but also complications that may result from recurrent percutaneous interventions.

## 1. Introduction

The treatment of iatrogenic and traumatic ureteral injuries is a rare entity encountered in the daily practice of interventional radiology. About 75% of all ureteric injuries are known to be iatrogenic and mostly occur during gynecological surgery (64%), colorectal surgery, urological procedures, and aortoiliac or aortofemoral bypass surgery [1]. It is crucial to identify ureteral injuries during the course of surgery; however, 50%–70% of them are missed in an acute setting [2]. Patients with missed injuries or delayed diagnosis often present with abdominal pain, fever, anuria, peritonitis, and even vaginal urinary leaking [3]. Urogenital injuries arise in 10% of patients with blunt and penetrating traumas [4]. Isolated injury of the ureter is extremely rare; thus, computed tomography (CT) is needed for the associated kidney, bladder, or bowel pathologies [5]. Radiology plays an important role in both the diagnosis of the injury and in guiding or carrying out the treatment [6].

There is no consensus about the treatment method for ensuring the integrity of the ureter. The choice of surgical or interventional radiological procedures depends on factors like diagnosis time, etiology, characteristics of the injury, the hemodynamic condition of the patient, and the experience of the center.

Percutaneous nephrostomy and simultaneous double-J (JJ) stenting under local anesthesia with ultrasound and fluoroscopy guidance is the treatment method of choice in interventional radiology, especially for partial injury of less than 50% of the ureter circumference. Use of a nephrostomy catheter and JJ stent allows for redirection of the flow and a chance for the injury to heal in 8–12 weeks [7]. 

The purpose of this study was to evaluate the safety, efficacy, and reliability of the external-internal ureteral catheterization technique using a single and patient-specific catheter, created manually for use in the treatment of ureteral injuries.

## 2. Materials and methods

### 2.1. Patients

A total of 14 patients who presented with iatrogenic and traumatic ureteral injury between May 2012 and January 2018, treated by external-internal catheterization technique in our interventional radiology unit, were included in the study. Patients with suspected ureteral injury were referred to our unit from the urology, gynecology, and general surgery departments primarily to visualize the injury site. Sonography, CT urography, and fluoroscopy were used as imaging methods to detect injury or related findings.

All of the data, including demographic information and clinical findings, were retrospectively obtained from the patients’ medical records and our procedure forms (Table).

**Table:** Patient demographics, ureteral injury, and catheterization-related data.

Patients		
Female	86%	n = 12
Male	14%	n = 2
Mean age (range)	44.29 years	(25–64 years)
Etiology		
Urology	57%	n = 8
Gynecology	36%	n = 5
General surgery	7%	n = 1
Injury site		
Proximal third	79%	n = 11
Middle third	7%	n = 1
Distal third	7%	n = 1
Proximal third + distal third	7%	n = 1
Mean ureteral length (range)	24.3 cm	(20–28 cm)
Pelvicalyceal dilatation		
None	57%	n = 8
Mild	14%	n = 2
Moderate	22%	n = 3
Severe	7%	n = 1
Mean duration of catheterization (range)	39 days	(15–87 days)

The ethics committee approved the study design and written informed consent was obtained from each of the patients.

### 2.2. Technique

Patients were primarily evaluated for the compliance of treatment. Inclusion criteria for the treatment were: patients with a hemodynamically stable condition and coagulation parameters within normal limits (INR <1.5, platelets >50,000/mm3), and those who could tolerate the prone position for percutaneous intervention and who had signed the consent form for procedure acceptance. Ultrasound (US; Loqic S6, GE Healthcare, USA) was used to detect renal dilatation and plan the procedure. All of the procedures were performed using US and fluoroscopy (Artis Zee, Siemens Healthcare, Germany) guidance under local anesthesia in a supine position.

In patients with no renal dilatation due to urine extravasation from the injury site, 2 ampoules of diuretics (Lasix, 20 mg/2 mL injectable ampoule, Sanofi Aventis, Turkey) were injected intravenously via 100 mL of 0.9% saline and the dilatation was followed by US. Subsequent to observing the adequate dilatation for percutaneous access, one more ampoule of diuretic was added and treatment began after sterile conditions were provided. Patients who received diuretics were monitored for possible hemodynamic changes. 

With the guidance of US, access was provided by using an 18-G needle in markedly dilated and 21-G needle in mildly or nondilated collecting systems, preferably from the upper or middle posterior calyx. Egress of the urine was seen after the stellate needle was removed, and the collecting system was made visible by contrast media under fluoroscopy. A J-tip Amplatz guidewire was advanced into the renal pelvis or proximal ureter through the needle. A 5-F, 65-cm Bern catheter (Boston Scientific, USA) was advanced into the proximal ureter over the guidewire in order to facilitate antegrade pyelography for detection of the injury level, such as occlusion, extravasation, or fistula if it existed. Using an 180-cm, 0.035-inch straight-tip hydrophilic coated guidewire, (Glidewire, Terumo, USA), effort was made to reach the bladder by passing the level of the injury in the ureter. In two patients, drainage of the urinoma and nephrostomy treatment were performed, primarily owing to proximal third ureteral injury accompanying urinoma. In 12 patients the guidewire and catheter were advanced into the bladder in the first session of treatment. After the confirmation of the bladder lumen by fluoroscopy, the lumen was filled with contrast media and the hydrophilic guidewire was exchanged with a stiff guidewire to provide support for the catheter. 

**Figure 1:**
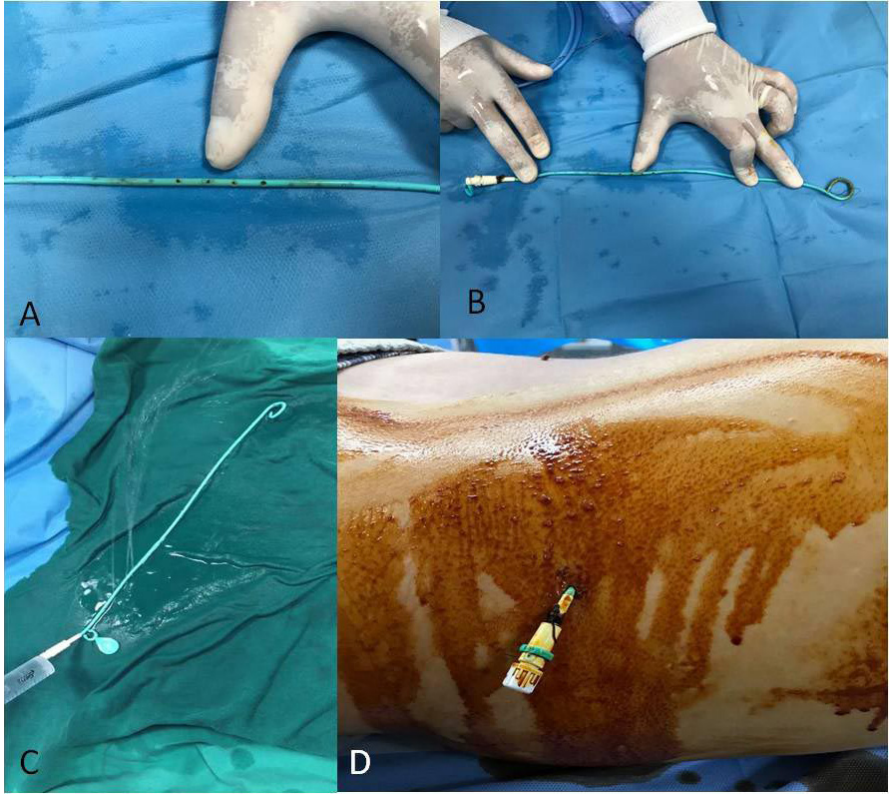
Holes (A) drilled manually at the proximal third of the 8-F, 35-cm pigtail drainage catheter (B), patency control of the holes with a saline injection (C), and the external part of the drainage catheter (D).

**Figure 2:**
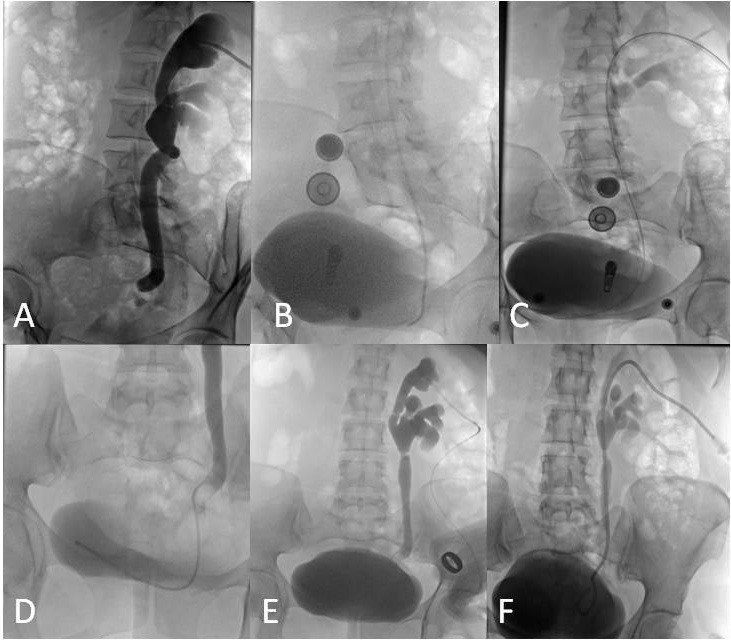
Blunt injury at the lower end of the ureter and pelvicalyceal dilatation due to cauterization during myomectomy surgery in a 33-year-old patient (A). Bern catheter advanced to the injury site (B) and hydrophilic guidewire reaching the bladder through the catheter (C). Lumen of the bladder filled by contrast media by the Bern catheter (D, E). The 8-F, 35-cm pigtail catheter advanced over the stiff guidewire to a position at the end of the pigtail in the bladder and manually drilled holes in the renal pelvis (F).

**Figure 3:**
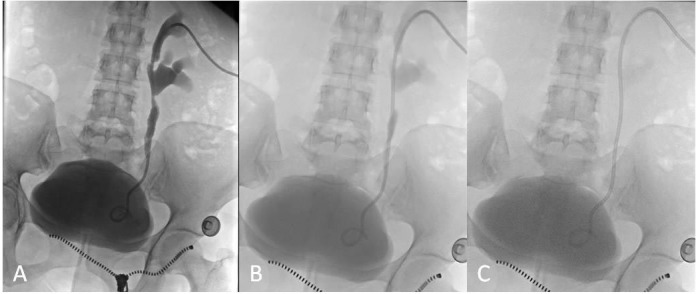
First day of follow-up for the same patient in Figure 2; pelvicalyceal system and bladder filled by contrast media (A), no blockage seen during drainage (B), and no contrast media remaining in the pelvicalyceal system (C).

At this stage, the external-internal catheterization technique was used to reduce the cost, using only one catheter for both internal and external drainage, and to overcome the exchange problems of the JJ when needed. For this purpose, the ureteral length of the patient was calculated using fluoroscopy images, while the stiff guidewire was advanced into the bladder to an optimum position. In accordance with the length of the ureter, 4 or 5 holes were drilled in the proximal third of an 8-F, 35-cm pigtail drainage catheter (Skater, Argon Medical Devices, USA), at a distance of 5 mm from each other, using the tip of a lancet (Figure 1). The catheter was advanced over the stiff guidewire to a position at the end of the pigtail in the bladder and at the manually drilled holes in the renal pelvis. Attention was paid to place the manually drilled holes in the pelvicalyceal system. Only in one patient was it noticed that the holes remained in the perirenal field and a new catheter was immediately exchanged for the existing stiff guidewire. When checking the antegrade pyelogram after catheter placement, the contrast media in the bladder and the pelvicalyceal system were viewed and no perirenal leakage was detected (Figure 2). The external side of the catheter was fixed to the skin with a suture and the cap of the catheter was closed to prevent risk of infection. Patients were called 1 day after the procedure to learn whether the catheter was working (Figure 3), and 1 month later to evaluate healing progress. 

At 1 month of follow-up, the stiff guidewire was advanced into the bladder through the catheter to maintain access and the catheter was removed. An introducer sheath was inserted over the wire and contrast media were injected in order to evaluate the track. The wire and introducer sheath were removed after passage into the bladder and no extravasation was detected. In the event of no healing in the ureter, a new external-internal catheter replaced the existing wire and the patient was called for follow-up 1 month later. Patients were followed by US after catheter removal.

## 3. Results

The study included 14 patients with ureteral injury (12 women and 2 men; mean age: 44.29 years, range: 25–64 years, SD: 13.69 years). The departments that the patients were referred from for treatment were urology, gynecology, and general surgery at a rate of 57% (n = 8), 36% (n = 5), and 7% (n = 1), respectively. The causes of ureteral injury were urological procedures for lithiasis (43%, n = 6), gynecological surgery (36%, n = 5), rectosigmoid surgery (7%, n = 1), penetrating injury (7%, n = 1), and partial nephrectomy (7%, n = 1). The most commonly affected segment was the distal third of the ureter at a rate of 79% (n = 11). Only in one patient was the coexistence of injury at the proximal and distal ureter revealed. This patient was referred to our hospital after a total rupture in both the proximal and distal ureter due to transurethral ureterorenoscopic lithotripsy. Ureteral anastomosis and hydronephrosis occurred following repair of the ureter in open surgery. Upon this, the decompression of the urinary system via nephrostomy was achieved and the external-internal catheterization technique was applied in the next session. The side of ureter injury was on the right in 8 patients (57%) and on the left in 6 patients (43%). Sonography performed before the procedure showed no dilatation of the collecting system in 8 patients (57%). Mild, moderate, and severe dilatation rates were 14%, 22%, and 7%, respectively. 

**Figure 4:**
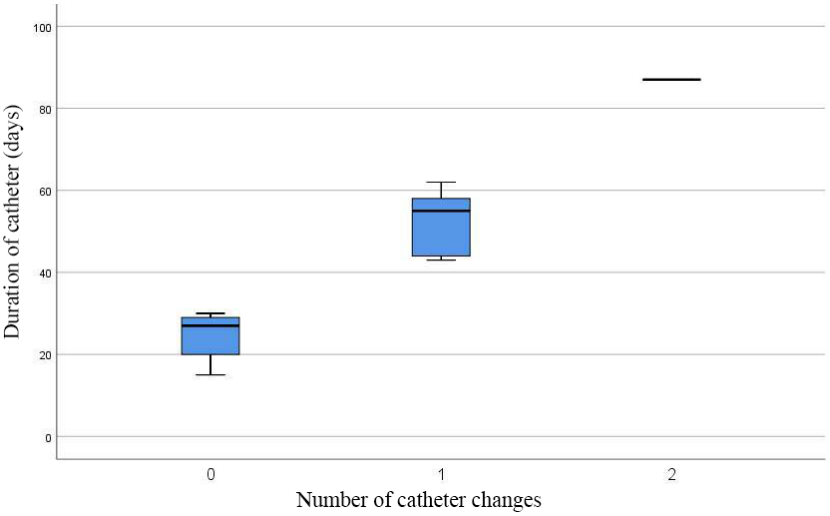
Relation between the duration of catheterization and number of catheter changes.

The mean duration of catheterization in the 8 patients with only one catheter was 27 days (range: 15–30 days, SD: 5.95), and in 5 patients with only one need of change it was 55 days (range: 43–62 days, SD: 8.50). Only in one patient was there a need to change the catheter twice during 87 days due to the complexity of the injury. The mean duration of catheterization in all of the patients was 39 days (range: 15–87 days, SD: 20.36). The relation between the duration of catheterization and number of catheter changes is summarized in Figure 4. 

The mean length of the ureter in 14 patients was 24.3 cm with a range of 20–28 cm (SD: 2.12). In 10 patients, the procedure was completed in the first session. In 2 patients urinoma drainage and in 4 patients nephrostomy was needed before the external-internal catheterization technique. The overall technical success was 100%. Partial failure was seen in only one patient, which occurred due to miscalculation of the ureter length and was fixed immediately with a new catheter. During and after the procedures, no major complications were encountered.

## 4. Discussion

The main problems with JJ stents are stent encrustation and migration, technical problems encountered due to the procedure or materials, postprocedural hematuria, and infection. The most common complication is pain related to trigone irritation, which occurs at a rate of 9%–13% [8–10]. Postprocedural hematuria was reported in different studies at a rate of 2%–21% [8]. Control nephrostomy is recommended for JJ patients, against the risk of occlusion owing to hematuria. One day after the procedure, the nephrostomy catheter is withdrawn upon visualization of the contrast material in the bladder in order to prove that the JJ stent is working. However, in patients with JJ stent dysfunction, a retrograde or antegrade approach may be required after withdrawal of the nephrostomy catheter. This might lead to additional interventions for patients and result in a cost burden for interventional radiology units. These all cause an increased risk of complications.

Infection is another complication of JJ stents, reported at a rate of 5%–10%, especially with long-term indwelling [8]. Conservative treatment is frequently sufficient; however, JJ removal has to be performed in the case of unresponsive fever and septicemia. In our patients, with the external-internal technique, the longest catheterization period did not exceed 3 months and no catheter-related infections were detected. Moreover, the catheter did not cause serious discomfort to the patients, because the external part of the catheter was not long, so the patients could tolerate it easily.

In the literature, it has been reported that the most affected segment in ureteral injuries is the distal third of the ureter, at a rate of 51%, followed by the proximal and middle thirds of the ureter at rates of 30% and 19%, respectively [3,11]. In our study, the most commonly affected segment was the distal third of the ureter at a rate of 79%.

However, the most common iatrogenic ureteral injuries reported in the literature were due to laparoscopic gynecologic surgeries; this finding differed in favor of urology in our study, by virtue of the lack of a gynecology department in our hospital [3].

Thanks to catheterization with this technique, in the case of a dilatation in the collection system or an increase in serum creatinine levels as a consequence of an occlusion of the distal part of the catheter, the catheter may function as nephrostomy with the aid of proximal holes until changing the catheter. In addition, better performance was observed during the procedures with this catheter in crossing the injury level and placement when compared with a JJ stent. Moreover, there was a significant decrease in the risk of malposition, defined as stent migration, which was reported with JJ stents at a rate of up to 16.3% [12].

We believe that when using this ureteral catheterization technique in patients with iatrogenic and traumatic ureteral injury, they will not need a JJ stent after recovery of the ureter. Furthermore, it is easy to apply and effective in reducing costs and complications due to recurrent percutaneous interventions in the event of occlusion and loss of access. Moreover, in patients with occlusion of the ureter due to malignancy, where the need for a JJ stent is lifelong, and considering that the need for a JJ stent exchange is every 4–6 months, exchange with this catheterization technique might be easier compared to a transurethral exchange. For this purpose, new studies are needed to observe the results with long-term use of external-internal catheters.

## References

[ref0] (2013). Ureteric injury: a challenging condition to diagnose and manage. Nature Reviews Urology.

[ref1] (2014). Management of iatrogenic ureteral injury. Therapeutic Advances in Urology.

[ref2] (2015). Iatrogenic urinary tract injuries: etiology, diagnosis and management. Seminars in Interventional Radiology.

[ref3] (2006). Imaging of genitourinary trauma. Scandinavian Journal of Urology and Nephrology.

[ref4] (1998). Ureteral trauma: preoperative studies neither predict injury nor prevent missed injuries. Journal of the American College of Surgeons.

[ref5] (2012). Contrast-material-enhanced MR urography in evaluation of postoperative lower urinary tract fistulae and leakages. Magnetic Resonance Imaging.

[ref6] (2011). The role of interventional radiology in urologic tract trauma. Seminars in Interventional Radiology.

[ref7] (2013). Indications and complications of double J ureteral stenting: our experience. Gomal Journal of Medical Sciences.

[ref8] (2007). Indications and complications of indwelling ureteral stenting at NMCH, Nawabshah. Pakistan Journal of Surgery.

[ref9] (2009). Dragan S. Long-term indwelling double-J stents: bulky kidney and urinary bladder calculosis, spontaneous intraperitoneal perforation of the kidney and peritonitis as a result of “forgotten” double-J stent. Vojnosanitetski Pregled.

[ref10] (2017). Interventional radiology in iatrogenic ureteral leaks: case series and literature review. La Radiologia Medica.

